# RNAi and Antiviral Defense in the Honey Bee

**DOI:** 10.1155/2015/941897

**Published:** 2015-12-21

**Authors:** Laura M. Brutscher, Michelle L. Flenniken

**Affiliations:** ^1^Department of Plant Sciences and Plant Pathology, Montana State University, Bozeman, MT 59717-3150, USA; ^2^Institute on Ecosystems, Montana State University, Bozeman, MT 59717-3490, USA; ^3^Department of Microbiology and Immunology, Montana State University, Bozeman, MT 59717-3460, USA

## Abstract

Honey bees play an important agricultural and ecological role as pollinators of numerous agricultural crops and other plant species. Therefore, investigating the factors associated with high annual losses of honey bee colonies in the US is an important and active area of research. Pathogen incidence and abundance correlate with Colony Collapse Disorder- (CCD-) affected colonies in the US and colony losses in the US and in some European countries. Honey bees are readily infected by single-stranded positive sense RNA viruses. Largely dependent on the host immune response, virus infections can either remain asymptomatic or result in deformities, paralysis, or death of adults or larvae. RNA interference (RNAi) is an important antiviral defense mechanism in insects, including honey bees. Herein, we review the role of RNAi in honey bee antiviral defense and highlight some parallels between insect and mammalian immune systems. A more thorough understanding of the role of pathogens on honey bee health and the immune mechanisms bees utilize to combat infectious agents may lead to the development of strategies that enhance honey bee health and result in the discovery of additional mechanisms of immunity in metazoans.

## 1. Introduction

Honey bees (*Apis mellifera*) contribute vital pollination services to agricultural crops and native landscapes, accounting for over $15 billion/year in economic value in the US [1]. In addition, the honey bee is a model organism for epigenetic, behavioral, and host-pathogen interaction studies [2–10]. Since 2006, the US and parts of Europe have experienced high annual colony losses (~33% annual loss in the US) [9, 11–13]. In the US, colony mortalities are partially attributed to Colony Collapse Disorder (CCD) [14–19]. These losses have stimulated greater interest in investigating honey bee biology, including the role of pathogens in colony mortalities and the role of the RNA interference (RNAi) mechanism in honey bee antiviral defense.

Pathogen incidence and abundance have been positively associated with CCD-affected colonies in the US [15, 19, 20] and colony losses in the US [21, 22], Canada [23], and European countries, including, Spain [24], Italy [25, 26], Belgium [27], and Germany [28]. In the US, Israeli acute paralysis virus (IAPV) was more abundant in colonies with less food stores and less developing bees/brood [29], and Lake Sinai viruses (LSV) 1 and 2 were more abundant in weak/less populated colonies from a small sample cohort [22]; however, this correlation was not seen in a larger sample cohort [30]. Honey bees are infected by a wide variety of pathogens (i.e., viruses, bacteria, microsporidia, and trypanosomatids) and also suffer from ectoparasitic mite (*Varroa destructor*) infestation (reviewed in [31]). The majority of honey bee pathogens are positive sense, single-stranded RNA viruses. The short-interfering RNA (siRNA) pathway of RNA interference (RNAi) is a major antiviral immune mechanism in solitary insects (reviewed in [32]) and is involved in honey bee antiviral defense.

RNAi is a post-transcriptional, sequence-specific, gene regulation mechanism conserved across several phyla, including plants, invertebrates, and mammals (reviewed in [33]). RNAi-mediated gene knockdown is a useful tool for assessing gene function in honey bees and other organisms for which additional reverse genetic tools are not available. While experimental introduction of virus sequence-specific dsRNA reduced honey bee virus infections in adults and larvae [29, 34–39], introduction of non-sequence-specific dsRNA also resulted in virus reduction and altered gene expression [38]. This is consistent with global changes in honey bee gene expression, including untargeted genes (i.e., off-target effects), observed from administration of dsRNA [40, 41]. Together these results suggest that dsRNA not only serves as the substrate for RNAi-mediated gene regulation but also may function as a trigger of gene regulatory signal transduction cascades. Future studies aimed at determining the relative contribution of RNAi and other honey bee antiviral defense pathways will be important for the development of strategies that limit virus infection and for investigating immune gene function in bees. In this review, we discuss RNAi as a tool for gene knockdown in honey bees, the role of the siRNA pathway of RNAi in honey bee antiviral defense, and additional honey bee antiviral defense pathways, including evidence of a non-sequence-specific dsRNA-stimulated immune pathway in honey bees.

## 2. RNA Silencing: Machinery and Functions

**Figure 1:**
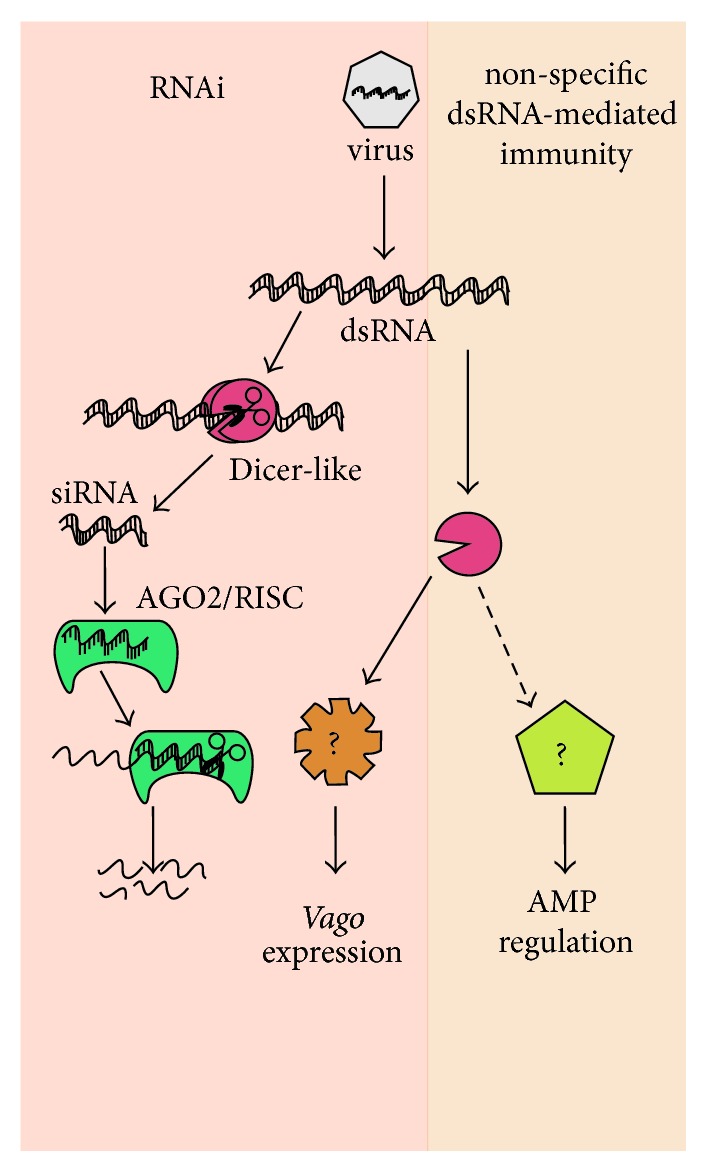
RNAi and non-sequence-specific dsRNA mediated antiviral defense in honey bees. The short-interfering RNA (siRNA) pathway of RNA interference (RNAi) is important for honey bee antiviral defense and experimental gene knockdown. Although not yet fully characterized, the honey bee RNAi-pathway is likely induced by* Am* Dicer-like cleavage of viral dsRNA into 21-22 bp siRNAs. Following cleavage, siRNA is bound by AGO2 (Argonaute-2), the catalytic subunit of the multiprotein RISC (RNA-induced silencing complex). The passenger strand is then released and the guide strand aids RISC in targeting and cleaving complementary viral genome sequences. In* Drosophila melanogaster*, Dicer-2 also acts as a dsRNA sensor which, when bound to dsRNA, initiates a signal transduction cascade that results in increased expression of* Dm* Vago and, in turn, increased expression of Jak-STAT pathway-associated genes. In honey bees, nonspecific dsRNA-mediated reduction in virus abundance [38] may involve* Am* Dicer-like and* Am* vago, but the mechanism(s) of this response have not been fully characterized. This figure is adapted from [10].

RNA silencing is a mechanism of post-transcriptional gene regulation conserved across several phyla that encompasses three distinct pathways (reviewed in [32, 42]), including the short-interfering RNA (siRNA), microRNA (miRNA), and piwi-interacting RNA (piRNA) pathways. Each of these pathways is characterized by its unique biological function and involvement of distinct proteins. The siRNA pathway is involved in antiviral defense in plants and invertebrates, but its function in mammalian immunology is debated (reviewed in [43–45]). This pathway is triggered by cytosolic dsRNA produced by replicating viruses or introduced experimentally. Double-stranded RNA is recognized and cleaved by the RNAse III enzyme, Dicer (Dicer-2 in* Drosophila* [46, 47] and Dicer-like in* Apis mellifera* [48]), into 21-22 bp short-interfering RNAs (siRNAs) (reviewed in [49]) (Figure 1). siRNAs are short dsRNAs with 5′ monophosphate ends and two nucleotide overhangs at their 3′ hydroxyl-termini (reviewed in [50]). The siRNAs are subsequently bound by Argonaute (AGO2), an endoribonuclease and catalytic component of the multiprotein RNA-induced silencing complex (RISC). One strand of the siRNA, the passenger strand, is then released, leaving the other strand, the guide strand, to target complementary viral and transposon sequences for cleavage (reviewed in [50]). miRNAs are derived from endogenous nuclear-encoded short-hairpin RNAs that are processed into shorter hairpin RNAs (pre-miRNA), cleaved by Dicer into 21-22 bp segments in the cytosol and incorporated into RISC (reviewed in [50]). The miRNA-containing RISC then targets complementary host-encoded mRNA transcripts for degradation or translational inhibition. Conversely, miRNAs can serve to induce transcription and translation of mRNA, reduce nonsense-mediated RNA decay, and improve mRNA stability (reviewed in [51]). miRNAs can function in antiviral response via targeting of viral nucleic acid and host gene regulation (reviewed in [51]). piRNAs, which are larger than siRNAs and miRNAs (24–32 nucleotides (nts)), are generated in a Dicer-independent manner from single-stranded RNA precursors transcribed from genomic regions (reviewed in [49, 52, 53]). piRNAs are involved in transposon silencing, epigenome regulation, and antiviral defense (reviewed in [49, 52, 53]). The focus of this review is on the use of the siRNA/RNAi pathway in experimental gene knockdown and its role in antiviral defense in honey bees. Further use of the term “RNAi” in this review is in regard to the siRNA pathway.

## 3. RNAi-Mediated Knockdown of Endogenous Gene Expression

RNAi is involved in antiviral defense and endogenous gene regulation. This mechanism can also be triggered experimentally to study gene function in organisms for which the tools for facile gene knockout are not available. RNAi-mediated gene knockdown has been used to study gene function in different honey bee developmental stages, including embryos [54–60], larvae [3, 56, 61–68], pupae [56, 69, 70], and fully developed adult honey bees [2, 4, 71–83] (reviewed in [84]). Importantly, these studies have demonstrated that the RNAi machinery is functional in honey bees.

The variable efficacy of RNAi-mediated gene knockdown observed in honey bees is likely gene-, dsRNA trigger-, and tissue-specific. The most effective use of RNAi-mediated gene knockdown in adult bees has been targeting of the hemolymph (insect blood) protein vitellogenin; vitellogenin expression was reduced at the mRNA (>75%) and protein levels in several studies [2, 72–74, 76, 81]. The efficacy of vitellogenin RNAi-mediated knockdown in honey bees is likely, in part, a consequence of its involvement in a positive regulatory feedback loop in which vitellogenin and juvenile hormone mutually suppress each other [72, 85]. In short, bees with less vitellogenin produce less vitellogenin. RNAi-mediated knockdown of vitellogenin in several studies has provided evidence for its role in aging by acting as an antioxidant [73] and in the timing of foraging behavior [74, 76]. The high efficiency of RNAi-mediated knockdown of honey bee vitellogenin may be partially attributed to enhanced targeting of siRNAs and dsRNAs to the fat body where vitellogenin is produced [86]. Similarly, intra-abdominal injection of either dsRNA or siRNA targeted against the* glycerol-3-phosphate dehydrogenase* (*amGPdh)* gene significantly decreased* amGPdh* transcripts in the fatty body (i.e., >70% decrease), but not in the ovaries, flight muscles, or head [78]. For comparison, a study that aimed to silence octopamine receptor expression via injection of dsRNA into the antennae observed ~40% decreased expression [71]. In addition, silencing of the hypopharyngeal amylase via dsRNA injection into the abdomen resulted in ~30% decreased expression in the hypopharyngeal glands [87]. The fat body is the site for insect humoral immunity (e.g., antimicrobial peptide production) and is involved in many metabolic processes (reviewed in [86, 88]). Therefore, gene knockdown studies in the fat body may be useful for studying honey bee metabolic and immune pathways.

## 4. RNAi in Antiviral Defense

The siRNA/RNAi is a major antiviral defense mechanism in solitary insects, including fruit flies and mosquitos (reviewed in [89]). The piwi-interacting RNA (piRNA) pathway has also been linked with antiviral response in insects via the detection of piRNA-sized viral RNAs in persistently infected* Drosophila* ovarian sheath cells and the discovery of ping-pong dependent piRNAs in arbovirus-infected* Aedes* spp. (reviewed in [49, 90]). miRNAs may have contrasting roles in insect-virus infection; virally derived miRNAs can disrupt host cell transcription and translation, but host miRNAs may be used to target and disrupt viral nucleic acid (reviewed in [51]). However, the roles of the miRNA and piRNA pathways in honey bee antiviral defense are not well characterized; thus, in this review, we focus on the siRNA pathway of RNAi.

The honey bee genome encodes the siRNA/RNAi machinery* dicer-like*,* ago-2*, and* r2d2* [48, 91], and bees are readily infected by positive sense, single-stranded RNA viruses (reviewed in [92]). These single-stranded RNA viruses generate double stranded RNA intermediates during their replication cycle and likely have significant secondary RNA structure within their genomes [92, 93], either of which may serve as Am Dicer-like substrates and trigger the honey bee siRNA pathway (Figure 1).

The role of RNAi in honey bee antiviral defense was first demonstrated when bees fed Israeli acute paralysis virus (IAPV) and IAPV-specific dsRNA had reduced IAPV levels as compared to bees fed only virus [34]. In addition, IAPV-specific siRNAs were detected by Northern blot analysis in the IAPV-specific dsRNA treated bees [34], indicating that the dsRNA and virus genomes were cleaved by Dicer-like and/or AGO2. IAPV replication was also decreased in bees fed siRNAs targeting the Internal Ribosomal Entry Site (IRES) of IAPV [39]. Similar results were obtained when larvae and adult bees fed dsRNA targeting Deformed wing virus (DWV) had reduced mortality, virus load, and deformed wing symptoms [37]. The effect of dsRNA administration on the outcome of virus infection was also examined in the Eastern honey bee,* Apis ceranae* [36]. In turn, there has been commercial/agricultural interest in utilizing RNAi-mediated antiviral treatments in honey bee colonies (reviewed in [35, 94, 95]). Initial field studies suggested that feeding honey bees IAPV-specific dsRNA resulted in increased honey production and larger colony size [35]; however, additional research is needed to confirm the mechanism of action and further investigate additional biological effects of dsRNA/siRNA treatments.

RNAi-mediated antiviral defense in naturally infected bees, which were not fed either dsRNA or siRNA triggers, was documented and characterized by sequencing small RNA libraries [96]. Small RNA sequence data indicated that bees from CCD-affected colonies had higher amounts of 22 nt siRNAs spanning the genomes of IAPV, Kashmir bee virus (KBV), and Deformed wing virus (DWV) as compared to non-CCD colonies. Most of the IAPV-specific siRNAs were negative sense, indicating their role as guide strands that target RISC to viral genomes/mRNAs [96]. Moreover, DWV virus levels were broadly proportional to the abundance of DWV-specific siRNAs in both orally infected and mite-vectored infections in developing bees [97]. Transcriptome (RNASeq) sequence data also indicated the role of the RNAi machinery in antiviral defense, as the expression of* Argonaute-2* and* Dicer-like* were greater in bees experimentally infected with IAPV as compared to mock-infected controls [6]. Intriguingly, transcriptional level regulation of the* Drosophila* RNAi genes in response to virus infection has not yet been documented [98, 99], suggesting that regulation of antiviral defense mechanisms in honey bees and fruit flies may differ.

Like many insect-infecting viruses, some honey bee viruses have likely evolved specific mechanisms to counteract RNAi-mediated antiviral defense, including virus-encoded suppressors of RNAi (VSR). For example, the B2 protein dimer of Flock house virus binds dsRNA, subsequently preventing Dicer-2 cleavage of long dsRNA [100, 101] and siRNA loading into RISC [101]. Dicistroviruses encode protein 1A, a VSR with differential modes of action (e.g., it binds to Dicer-2 or AGO2) and efficacy that varies by virus (reviewed in [90]). Based on analysis of VSR-expressing viruses (i.e.,* Drosophila* C virus and Cricket paralysis virus), the presence of the highly conserved DvExNPGP motif and upstream coding sequences are indicative of the ability to express VSR proteins [102, 103]. Sequence analysis revealed that the honey bee dicistroviruses IAPV and KBV and Acute bee paralysis virus (ABPV) contain a DvExNPGP motif at the 5′ terminus of their genomes, suggesting these honey bee-infecting viruses may encode a VSR [29]. Experimental feeding of naturally IAPV-infected bees with siRNAs targeting the putative IAPV-encoded RNAi suppressor decreased IAPV loads at least three times more than treatment with siRNAs targeting the IAPV IRES [29, 39]. Better understanding of the importance of RNAi in honey bee antiviral defense and the means by which viruses may evade the honey bee antiviral response will facilitate the manipulation of these mechanisms in the lab as well as their potential application in the field setting.

The dsRNA uptake mechanisms in insects and their relationship to systemic RNAi and antiviral defense are not completely understood. Current studies suggest that there are at least two mechanisms of dsRNA uptake in insects: transmembrane channel-mediated uptake (reviewed in [104]) and endocytosis-mediated uptake [105–107]. SID-1 (systemic RNA defective), a dsRNA-transporting transmembrane protein originally identified in* C. elegans* [108, 109], has been implicated in facilitating systemic RNAi in honey bees; bees injected with dsRNA had over three times greater expression of SID-1 than controls [110].* C. elegans* also encodes additional SID proteins, SID-2, SID-3, and SID-5, which have also been implicated in dsRNA uptake but have not been identified in the honey bee genome [111–113]. Honey bees encode for one SID-1 ortholog with two protein isoforms (XP_006565236.1 and XP_006565237.1), which both share ~25% amino acid identity with the* C. elegans* SID-1 (NP_504372.2) [114]. In addition, transgenic* Drosophila* S2 cells expressing the* C. elegans* SID-1 protein had improved dsRNA uptake [109]. Interestingly, SID-1 is not present in all insect genomes (reviewed in [104]), including* Drosophila* [115], and is not required for systemic RNAi in locusts [116]. Proteins involved in phagocytosis and endocytosis may function in dsRNA uptake, as the scavenger receptors SR-CI and Eater and the endocytosis-associated proteins clathrin heavy chain and H+ ATPase are important for dsRNA uptake in* Drosophila* S2 cells [105, 106]. Investigating dsRNA uptake and systemic RNAi will be an important step towards further characterizing honey bee antiviral response.

Double-stranded RNA treatment of honey bees has also been employed to reduce gene expression in honey bee-associated parasites including the microsporidia* Nosema ceranae* [117] and the ectoparasitic mite* Varroa destructor* [118]. Honey bees inoculated with* Nosema* spores and fed dsRNA targeting* Nosema*-specific ADP/ATP genes had reduced* Nosema* spore count, and* Nosema* had lower expression of the targeted genes [117]. Likewise, when bees were fed dsRNA targeting mite sequence-specific housekeeping genes, mites had lower levels of the targeted transcripts [118]. Interestingly, long, unprocessed dsRNAs were detected in bee hemolymph three days after feeding dsRNA [118]. The biological relevance of dsRNA as a systemically active molecule in naturally infected honey bees is unknown, but it is remarkable that orally introduced dsRNA remains stable enough to spread throughout the honey bee host and into associated parasites [117, 118].

It is interesting that several studies have demonstrated that dsRNA/siRNA feeding is an effective strategy to reduce virus loads in both larval-stage and adult bees, while achieving effective* in vivo* gene silencing is difficult in mammalian model systems (reviewed in [119]). Tail-vein injections of siRNA in postnatal mice have been an effective strategy for gene knockdown [120], but overall systemic siRNA delivery into mammalian systems often requires siRNAs with chemical modifications such as lipophilic conjugates or nanoparticle mediated delivery (reviewed [119, 121]). Preliminary results on the effects of RNAi-mediated treatment of honey bee viruses and parasites are promising, but additional investigation is required to better understand the feasibility, effectiveness, and risk of off-target effects. Additionally, it will be important to develop methods to functionally test the role of the RNAi machinery via gene knockout/knockdown. Genome integration of IAPV also requires further examination [122]. Both genome-integrated RNA viral sequences, putatively encoding for target nucleic acid or reverse-transcriptase, and RNAi are involved in limiting and maintaining persistent virus infections in* D. melanogaster* [42, 107]. These and other studies will reveal the role of RNAi in honey bee antiviral defense.

## 5. Additional Antiviral Defense Mechanisms

Multiple mechanisms are involved in insect immune responses, including phagocytosis, melanization, and signal transduction of the Toll, Imd (immune deficiency), and Jak/STAT (Janus kinase and Signal Transducer and Activator of Transcription) innate immune response pathways which result in the production of antimicrobial peptides (AMPs) and other effector proteins (reviewed in [89, 90, 123]). There are multiple orthologous proteins utilized by both insect and mammalian immune pathways (reviewed in [124, 125]), including Toll-like receptors (TLRs). While mammalian TLRs recognize and bind specific pathogen associated molecular patterns, the* D. melanogaster* Toll acts downstream of pathogen recognition [90, 123]. Honey bees encode for all the major components of the Toll, Imd, JNK, Tor, and Jak-STAT pathways (except* upd*), AMPs (i.e.,* abaecin*,* hymenoptaecin*,* apidaecin*, and* defensin*), and prophenoloxidases [126].

Transcriptional studies of virus-infected honey bees have implicated the Jak-Stat, Toll, and Imd pathways in antiviral defense (reviewed in [10]). For example, bees infected with DWV had greater expression of the Imd pathway member* dorsal-1A* [25], and bees fed IAPV had increased expression of Toll pathway members (i.e,* toll-6*,* cactus*, and the AMP* hymenoptaecin*) and Jak/STAT pathway members (i.e.,* cbl*,* stat*,* pias*, and* hopscotch*) [29]. Nevertheless, not all infection studies investigating transcriptional responses to the same virus followed the same trends. In contrast to bees fed IAPV, bees from naturally infected IAPV colonies did not have differential regulation of Jak/STAT or Imd pathways [29]. These inconsistencies may be due to different experimental factors such as difference in virus isolate/strain utilized, infection route, age of bees, and tissue examined (reviewed in [10]). Further investigation of the role of these and other innate immune pathways in honey bee antiviral defense will lead to a better understanding of the mechanism(s) of honey bee antiviral defense and reveal unique honey bee host-virus interactions.

## 6. Nonspecific dsRNA Triggered Virus Reduction

In addition to inducing RNAi, dsRNA may also engage a previously uncharacterized non-sequence-specific immune pathway in honey bees [38] (Figure 1). Bees coinjected with Sindbis virus (SINV) and sequence-specific dsRNA or non-sequence-specific dsRNA had similarly decreased viral titers as compared to bees injected with virus only [38]. Likewise, adult bees treated with non-virus specific dsRNA (i.e., GFP- (green fluorescent protein-) targeting) and infected with DWV had a greater rate of survival as compared to DWV-infected bees that received no dsRNA-treatment [37]. In addition, experimental introduction of nonspecific dsRNA alone in honey bees perturbs honey bee gene expression [38, 41]. Transcriptome analysis of honey bee larvae fed GFP-targeting dsRNA revealed ~1,400 differentially regulated genes (DEGs) [41]. Nine genes had sequence similarity with 21 nt regions of GFP, indicating off-target RNAi [41]. However, most DEGs did not share sequence similarity with dsRNA-GFP and were reported to function in oxidoreductase activity, aging, cell homeostasis, morphogenesis, response to external stimulus and stress, and immune response [41]. Also, bees injected with non-sequence-specific dsRNA had differential expression, including decreased expression of several* apidaecin *AMP family members [38]. In a recent study that examined the role of RNAi-mediated antiviral defense in bumblebees (i.e.,* Bombus terrestris*), adults fed non-sequence-specific dsRNA had increased survival when infected with IAPV and similar virus titers as compared to bees fed dsRNA targeting IAPV [127]. Together these results suggest that honey bees and other members of the* Apidae* family may have an alternative dsRNA-stimulated immune pathway akin to the interferon response in mammals. Mammals have dsRNA recognition receptors such as Toll-like receptor 3 (TLR3), Protein kinase R (PKR), Retinoic acid-inducible gene 1 (RIG-I), and Melanomadifferentiation-associated gene 5 (MDA-5), that when activated, induce expression of numerous genes that contribute to an antiviral state (reviewed in [128]). Analogously, non-sequence-specific dsRNA-mediated immune pathways may be important for antiviral defense in honey bees.

Nonspecific dsRNA-triggered antiviral immunity has also been observed in other arthropods including Chinese oak silk moth pupae [129], shrimp [130–134],* Bombyx mori* larvae [135], and sandfly cells [136], implicating dsRNA as a viral pathogen associated molecular pattern (PAMP or VAMP). In addition, there is evidence that Dicer-2 serves as a pathogen recognition receptor (PRR) of dsRNA in both* D. melanogaster* [47] and* Culex pipiens* f.* molestus* mosquito cells [137]. When bound with dsRNA, Dicer-2 stimulates a signal-transduction cascade that results in increased expression of* vago* and Jak-Stat pathway genes (reviewed in [90, 123]) (Figure 1). Intriguingly, larvae orally infected with DWV from* Varroa* infested colonies had significantly greater expression of the honey bee ortholog of* vago* as compared to control larvae from colonies with lower mite pressure [97]. Though administration of non-sequence-specific dsRNA does not always improve survival or reduce viral titer in virus infected bees [34, 36], it is important to further examine the mechanisms involved in non-sequence-specific dsRNA-mediated antiviral immunity in honey bees.

## 7. Conclusion

Honey bees are essential pollinators of agricultural crops and many plant species. Since 2006, annual losses of honey bee colonies in the US have been high (i.e., averaging 33%). Pathogen incidence and abundance correlate with CCD, as well as colony health and loss in multiple studies. Continued investigation of honey bee host-pathogen interactions is important to better understand the role of pathogens in colony losses. Many positive sense single-stranded RNA viruses infect honey bees. Honey bee virus infections result in a range of outcomes, likely caused by varying immune responses due to genetic differences [138, 139], coinfection with additional pathogens [20, 31, 140], adequate bee nutrition [141–143], the effect of the bee microbiome [144, 145], and/or exposure to environmental factors including agrochemicals and weather events [146–149]. The RNAi mechanism plays a role in honey bee antiviral defense but the relative contribution of this and other immune pathways has not been fully elucidated. The efficacy of RNAi-mediated treatment against honey bee viruses together with the fact that honey bee viruses encode for putative VSRs supports that RNAi is an important honey bee antiviral defense mechanism. In addition, several studies implicate the involvement of innate immune pathways (i.e., Jak-Stat, Toll, and Imd) and non-sequence-specific dsRNA-mediated immune responses in honey antiviral defense.

Honey bee gene knockout models are not yet available, so experimental induction of RNAi has become an important tool for studying gene function in honey bees. Although effective RNAi-mediated gene knockdown has been demonstrated in the fat body, gene knockdown in other tissue types (e.g., reproductive tissue [78]) remains a challenge. Further development of honey bee cell culture systems [150–153], and perhaps the use of the endoribonuclease CRISPR/Cas9-mediated gene knockout system [154], will also facilitate future investigations of RNAi and innate immune pathways in honey bees. Continued investigation of honey bee host-pathogen interactions and better characterization of the honey bee immune system may result in implementation of strategies that benefit honey bee colony health and result in the discovery of additional evolutionarily conserved immune mechanisms.
